# A Cross-Sectional Study to Assess Street Food Vendors’ Adherence to the Bureau of Indian Standards (BIS) in an Urban Slum of Pune, India

**DOI:** 10.7759/cureus.80457

**Published:** 2025-03-12

**Authors:** Nitin Kaushal, Puja Dudeja, Sanjay Chaturvedi, Sunil Thakur, Poonam Khanna, Poojan Marwaha, Sushruti Kaushal

**Affiliations:** 1 Community Medicine and School of Public Health, Postgraduate Institute of Medical Education and Research, Chandigarh, IND; 2 Community Medicine, Armed Forces Medical Services, Jaipur, IND; 3 Community Medicine, Armed Forces Medical Services, Pune, IND; 4 Anesthesiology, All India Institute of Medical Sciences, Bilaspur, Bilaspur, IND; 5 Obstetrics and Gynecology, All India Institute of Medical Sciences, Bilaspur, Bilaspur, IND

**Keywords:** demographic, employment, food industry, food safety, low- and middle-income countries

## Abstract

Background

Street food is widespread in low- and middle-income countries like India, offering nutrition and jobs to many. Being in the informal sector can also pose foodborne illness risks due to limited access to clean water, sanitary facilities, and food safety knowledge. A cross-sectional study can identify the prevalence of unsafe practices and provide a snapshot of the current state of food safety and hygiene practices among street vendors. To prevent such outbreaks, the Bureau of Indian Standards (BIS) established standards for street food vendors under the Food Safety and Standards Act 2006.

Method

This cross-sectional study aims to assess the conformance of street food vendors with the BIS requirements for food safety and various factors affecting the same, assuming that 50% of street food vendors would conform to the requirements. The prevalence was assumed to be 50% as no study was available for reference for conformance with the BIS requirements. With a precision of 10% on either side of the truth and with 95% confidence to estimate the proportion of street vendors conforming with the BIS requirements, a sample size of 97 was calculated. The street food vendors operational in the area were listed in an urban slum of Pune in Maharashtra, and 100 among them were selected through a computer-generated random number table. A questionnaire based on the BIS was developed, with a minimum score of 0 and a maximum score of 114. The investigator interviewed each vendor for 45-60 minutes using a structured questionnaire. The questionnaire was validated by conducting a pilot study in the same area with a sample size of 20 vendors.

Results

The vendors' scores were evaluated against various demographic variables, including age, experience, education, place of residence, and monthly income. The data collected was analyzed for descriptives, and categorical data was analyzed using Chi-square and Fisher’s exact statistical tests using IBM SPSS Statistics for Windows, Version 20 (Released 2011; IBM Corp., Armonk, New York, United States). The mean age of the vendors was 30.5 ± 8.06 years, with an average experience of 4.87 ± 2.93 years. The average monthly income of the vendors was Rs 6004 ± 3179, and the majority of vendors were males (95%). Of the vendors, 64% scored satisfactory, with an overall score of ≥50%. The vendors' mean score was 60.2 ± 13.9. Reasons for the poor score were related to waste disposal techniques, availability of ample water for various activities, facilities for refrigeration, proper usage of gloves, pest control activities, and lack of formal training. The study observed a significant relationship between place of residence and overall score (p = 0.002) and between monthly income and overall score (p = 0.023).

Conclusion

The street food industry plays a vital role in meeting people's food requirements and the nation's economic structure, employing many people. Providing safe and hygienic street food to consumers is an important aspect of the industry, and various factors interplay in its complex mechanism. In this study, waste disposal, pest control, and lack of training were important factors causing low scores for street food. These factors can be rectified by the coming together of municipal bodies to provide earmarked locations, training and waste disposal facilities, water and electricity department, health department for regular hygiene inspections, and law enforcement department to implement the above points.

## Introduction

An estimated 2.5 billion people around the world consume street food daily, owing to its ease of availability and economic nature [1]. It is also estimated that 20%-25% of expenditure on food in developing countries is incurred outside the home [2]. Street-vended food, defined as the food supplied by vendors for immediate consumption or later use without further processing or preparation, is a significant part of the urban food supply for two-thirds (74%) of the WHO member states [3]. A wide variety of foods are vended on the streets depending upon the residents' taste preferences and socioeconomic status. In India, a developing country with a population migrating from rural to urban areas in search of education and work, street food serves as a major source of food for millions of people in metropolitan cities [4]. Moreover, street foods have gained more popularity, with working women having less time for household activities. Street vendors provide inexpensive, convenient, and nutritious food to a large population in the modern day [5]. In a study conducted in Kenya, it was observed that street foods are a potential source of various micronutrients like zinc, iron, and vitamin A [6]. On the other hand, street food is also a source of self-employment for many [3]. Street food is sold in busy public areas like pavements, school premises, beaches, and rail and bus stations on a stand, cart, or kiosk [7].

The downside to this easy availability is that unhygienic food can lead to foodborne illnesses and, in extreme events, can even lead to the death of the consumer [8]. According to a WHO report, one in 10 people fall ill every year from eating contaminated food, and 420,000 die as a result of foodborne infections throughout the world. The same report says the Southeast Asian region has the second-highest burden of foodborne illnesses per population and the highest in terms of sheer numbers [9]. In India, the Integrated Disease Surveillance Programme (IDSP) under the National Centre for Disease Control (NCDC) has reported more than 214 food poisoning outbreaks till the 31st week of 2024, and food poisoning is the second most common cause of outbreaks in the country [10].

Street food has implications for the health of consumers across the world. Various factors such as inadequate hygiene practices by food handlers, insufficient facilities of potable water and waste disposal, inadequate infrastructure, inadequate facilities for food storage (raw/cooked) which promote microbial growth, and exposure of food to animals such as rodents and insects are identified causes of rendering the street food unsafe [11]. In India, there is a lack of studies conducted on the food safety and hygiene practices of street food vendors, especially in the last five years. The Bureau of Indian Standards (BIS), the National Standard Body of India, which regulates the development of activities related to standardization, marking, and quality certification of goods, brought out the requirements from street food vendors for the provision of safe and hygienic food in 2012 [12]. Although the standards for street food vendors in 2012, the compliance of street food vendors with these requirements have not been brought out in scientific studies and largely remains an unexplored topic. Hence, this study was planned to assess whether street food vendors comply with these food safety requirements.

## Materials and methods

A cross-sectional study was conducted on 100 street food vendors in an urban slum of Pune in Western Maharashtra to check for the conformance of street vendors with the standards laid down by BIS and various factors affecting the conformance. The proportion of vendors conforming to BIS requirements was assumed to be 50% as no study was available for reference for conformance with BIS requirements. With a precision of 10% on either side of the truth and with 95% confidence to estimate the proportion of street vendors conforming with BIS requirements, a sample size of 97 was calculated; hence, 100 street food vendors were included in the study. The street food vendors operational in the area were listed in an urban slum of Pune in Western Maharashtra, and 100 among them were selected through a computer-generated random number table. A questionnaire based on the BIS guidelines was developed, with a minimum score of 0 and a maximum score of 114. Each vendor was interviewed by the same investigator for 45-60 minutes using a structured questionnaire at the site of the vending of food. The questionnaire was validated by conducting a pilot study in the same area with a sample size of 20 vendors. The minimum sample size required for a pilot study is 10% of the parent study; however, a sample of 20 was taken.

Vendors above the age of 18 years with a minimum experience of two years and willing to participate in the study were included in the list. Any food business operator with less than two years of experience or a permanent establishment was excluded from the study [13]. The street food vendors were numbered in the area and selected through a computer-generated random number table among those who qualified and consented to the study. The selected vendors were then interviewed one-on-one. The study was conducted for 1½ years (Jan 2016-Jul 2017), and the scores for each vendor were calculated. The questionnaire consisted of 12 domains, as per BIS guidelines [12]. A total of 107 subheads in these domains were identified and converted into scores. The 12 domains and number of subheads in each domain are as follows: (a) raw material, two subheads; (b) transportation, reception, and storage of raw materials, seven subheads; (c) vending location, 16 subheads; (d) vending cart, 16 subheads; (e) utensils and cutting tools, 13 subheads; (f) hygienic practices, 15 subheads; (g) personal hygiene and habits, 10 subheads; (h) food preparation, cooking and handling, seven subheads; (i) protection and serving of food, 14 subheads; (j) handling and disposal of waste, four subheads; (k) pest control, two subheads; and (l) Training on food safety, one subhead.

The data was collected from 100 street food vendors based on 12 variables outlined in the BIS 2012 guidelines. The scores obtained in each domain and the overall score were then converted into percentages. As there was no reference study that had converted the BIS requirements into a scoring questionnaire, it was assumed that vendors scoring less than 50% would be graded as unsatisfactory, while those scoring 50% and above would be graded as satisfactory. Data analysis was conducted using IBM SPSS Statistics for Windows, Version 20 (Released 2011; IBM Corp., Armonk, New York, United States). Fisher’s exact test and Chi-square test were applied to the categorical data, and a p-value less than 0.05 was taken as statistically significant. Ethical approval for the study was obtained from the Institutional Ethics Committee of the Armed Forces Medical College, Pune.

## Results

The data was collected from the sample for demographics, including age, gender, educational status, work experience, monthly income, and native place. The baseline demographic characteristics are shown in Table 1. None of the vendors had received any formal training (Tables 1-2).

**Table 1:** Sociodemographic characteristics I

Variable	Categories	Frequency	Percentage
Gender	Male	95	95%
	Female	05	05%
Age (years)	<25	29	29%
	25-34	43	43%
	35-44	21	21%
	≥45	7	7%
Experience (years)	<4	59	59%
	5-9	33	33%
	≥10	8	8%
Education	<10 standard	32	32%
	10-12 standard	56	56%
	≥12 standard	12	12%
Income	< Rs 5000	33	33%
	Rs 5000 – 9999	51	51%
	≥ Rs 10000	16	16%
Place of residence	Local	40	40%
	Migrant	60	60%
Formal training received	Yes	0	0%
	No	100	100%

**Figure 1:**
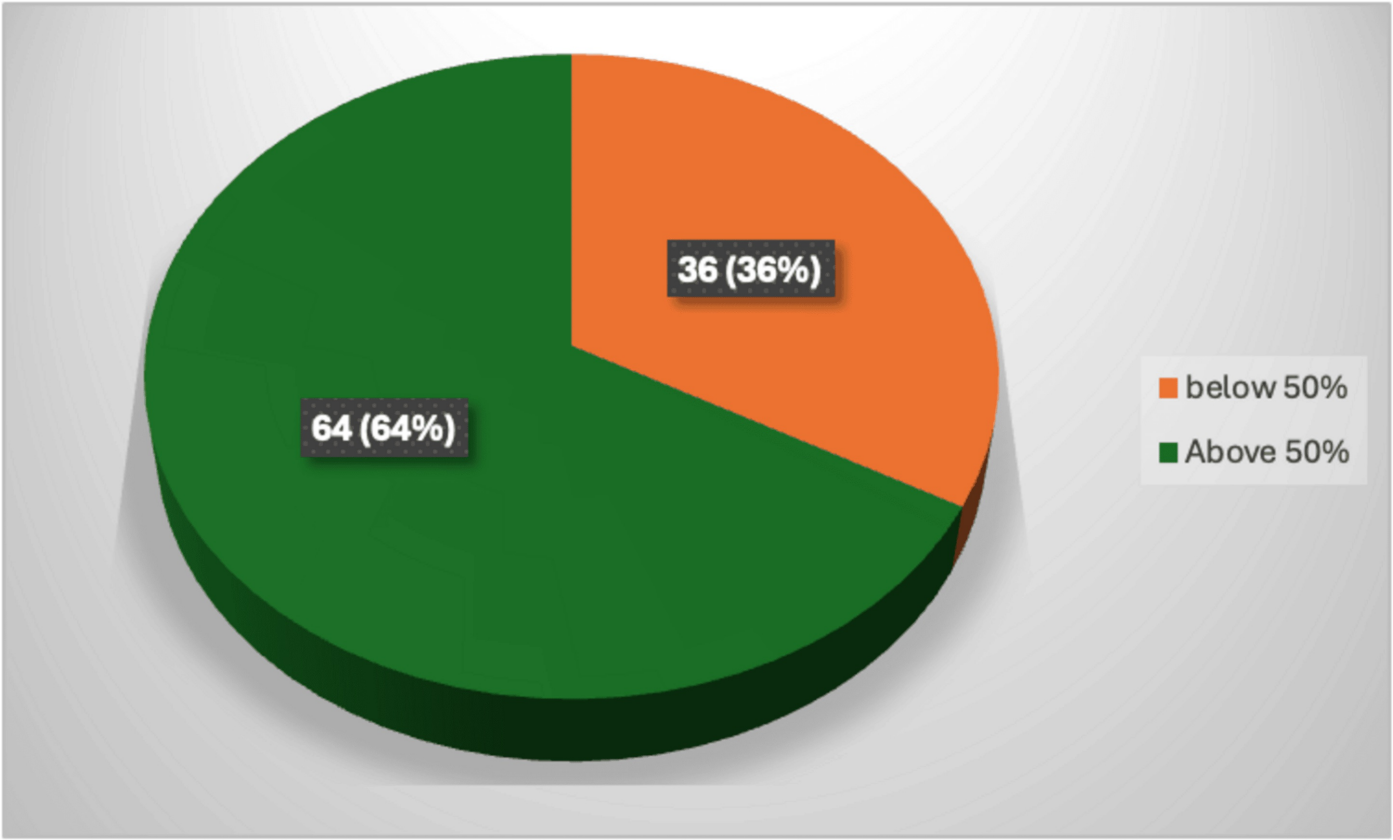
Percentage score of vendors

Based on the questionnaire developed from the BIS guidelines, the highest score obtained by the participants was 84%, and the lowest was 22.8%, with a mean score of 60.2 ± 13.9. A total of 64% of the vendors scored satisfactory in the overall score, while 36% of the vendors scored unsatisfactorily (Figure 1). Reasons for the poor score were related to waste disposal techniques like the use of covered rubbish bins, use of disposable plastic bags, etc.; water-related fields like handwashing facilities, washing utensils under running water, and availability of drinking water; and facilities for refrigeration and proper usage of gloves like discarding gloves during interruptions, washing hands before putting on gloves, not to use gloves for collecting money. The low scores were also related to a lack of pest control activities and a lack of formal training to the vendors.

**Table 2:** Sociodemographic characteristics II

	N	Minimum	Maximum	Mean	Std. deviation
Age (years)	100	18	55	30.5	8.06
Experience (years)	100	2	16	4.87	2.93
Income (Indian Rupee)	100	1200	15000	6004.00	3179

Relationship between demographic variables and variables as per the BIS guidelines

The study evaluated the overall scores of the vendors in relation to the demographic variables of the street food vendors. Place of residence and monthly income of the vendors were the demographic variables that had significant association with overall scores. It was observed in the study that the local vendors scored better than the migrant street food vendors, with a p-value of 0.002 in the Chi-square test. It was also shown in the study that the vendors who had higher monthly income had a better score with a p-value of 0.023 in the Chi-square test. In this study, the relation of the rest of the demographic variables, which are the age of the vendor, gender of the vendor, level of education, and experience of the vendor in years for street food vending, was not found to be statistically significant with the overall scores of the vendors. The BIS domains were also individually evaluated for association with the demographic variables of the street food vendors. The relationship of each variable with 12 domains is presented in the following tables (Table 3-8).

**Table 3:** Relationship between age groups and the various domains of the BIS guidelines BIS: Bureau of Indian Standards Score >50%: satisfactory; score ≤50%: unsatisfactory

Variable	Age group (age in years)	Number	Satisfactory N (%)	Unsatisfactory N (%)	p-value (Fisher’s exact test)
Raw material	<25	29	16 (55.2)	13 (44.8)	0.738
	25-34	43	29 (67.4)	14 (32.6)	
	35-44	21	14 (66.7)	7 (33.3)	
	>45	7	4 (57.1)	3 (42.9)	
Transportation, reception, and storage of raw material	<25	29	0 (0)	29 (100)	0.002*
	25-34	43	5 (11.6)	38 (88.4)	
	35-44	21	7 (33.3)	14 (66.7)	
	>45	7	2 (28.6)	5 (71.4)	
Vending location	<25	29	16 (55.2)	13 (44.8)	0.003*
	25-34	43	35 (81.4)	8 (18.6)	
	35-44	21	20 (95.2)	1 (4.8)	
	>45	7	7 (100)	0 (0)	
Vending cart	<25	29	12 (41.4)	17 (58.6)	<0.001*
	25-34	43	32 (74.4)	11 (25.6)	
	35-44	21	20 (95.2)	1 (4.8)	
	>45	7	6 (85.7)	1 (14.3)	
Utensils and cutting tools	<25	29	25 (86.2)	4 (13.8)	0.324
	25-34	43	40 (93)	3 (7)	
	35-44	21	21 (100)	0 (0)	
	>45	7	7 (100)	0 (0)	
Hygienic practices	<25	29	0 (0)	29 (100)	0.423
	25-34	43	3 (6.97)	40 (93.02)	
	35-44	21	2 (9.52)	19 (90.47)	
	>45	7	0 (0)	7 (100)	
Personal hygiene and habits	<25	29	29 (100)	0 (0)	0.212
	25-34	43	41 (95.3)	2 (4.7)	
	35-44	21	21 (100)	0 (0)	
	>45	7	6 (85.7)	1 (14.3)	
Food preparation, cooking, and handling	<25	29	2 (6.9)	27 (93.1)	<0.001*
	25-34	43	11 (25.6)	32 (74.4)	
	35-44	21	13 (61.9)	8 (38.1)	
	>45	7	5 (71.4)	2(28.6)	
Protection and serving of food	<25	29	11 (37.9)	18 (62.1)	0.108
	25-34	43	14 (32.6)	29 (67.4)	
	35-44	21	12 (57.1)	9 (42.9)	
	>45	7	5 (71.4)	2 (28.6)	
Handling and disposal of waste	<25	29	0 (0)	29 (100)	-
	25-34	43	0 (0)	43 (100)	
	35-44	21	0 (0)	21 (100)	
	>45	7	0 (0)	7 (100)	
Pest control	<25	29	0 (0)	29 (100)	-
	25-34	43	0 (0)	43 (100)	
	35-44	21	0 (0)	21 (100)	
	>45	7	0 (0)	7 (100)	
Training on food safety	<25	29	0 (0)	29 (100)	-
	25-34	43	0 (0)	43 (100)	
	35-44	21	0 (0)	21 (100)	
	>45	7	0 (0)	7 (100)	

**Table 4:** Relationship between gender of the vendors and the various domains of the BIS guidelines BIS: Bureau of Indian Standards Score >50%: satisfactory; score ≤50%: unsatisfactory

Variable	Gender (M/F)	Number	Satisfactory N(%)	Unsatisfactory N(%)	p-value (Fisher’s exact test)
Raw material	Male	95	58 (61.1)	37 (38.9)	0.154
	Female	5	5 (100)	0 (0)	
Transportation, reception, and storage of raw material	Male	95	13 (13.7)	82 (86.3)	0.537
	Female	5	1 (20)	4 (80)	
Vending location	Male	95	74 (77.9)	21 (22.1)	1
	Female	5	4 (80)	1 (20)	
Vending cart	Male	95	65 (68.4)	30 (31.6)	0.318
	Female	5	5 (100)	0 (0)	
Utensils and cutting tools	Male	95	88 (92.6)	7 (7.4)	1
	Female	5	5 (100)	0 (0)	
Hygienic practices	Male	95	4 (4.2)	91 (95.8)	0.230
	Female	5	1 (20)	4 (80)	
Personal hygiene and habits	Male	95	92 (96.8)	3 (3.2)	1
	Female	5	5 (100)	0 (0)	
Food preparation, cooking, and handling	Male	95	29 (30.5)	66 (69.5)	0.644
	Female	5	2 (40)	3 (60)	
Protection and serving of food	Male	95	38 (40)	57 (60)	0.158
	Female	5	4 (80)	1 (20)	
Handling and disposal of waste	Male	95	0 (0)	95 (100)	-
	Female	5	0 (0)	5 (100)	
Pest control	Male	95	0 (0)	95	-
	Female	5	0 (0)	5	
Training on food safety	Male	95	0 (0)	95	-
	Female	5	0 (0)	5	

**Table 5:** Relationship between education of the vendors and the various domains of the BIS guidelines BIS: Bureau of Indian Standards Score >50%: satisfactory; score ≤50%: unsatisfactory

Variable	Educational status (secondary, senior secondary, and above)	Number	Satisfactory N (%)	Unsatisfactory N (%)	p-value (Fisher’s exact test)
Raw material	<10	32	15 (46.9)	17 (53.1)	0.003*
	10-12	56	36 (64.3)	20 (35.7)	
	>12	12	12 (100)	0 (0)	
Transportation, reception, and storage of raw material	<10	32	2 (6.3)	30 (93.7)	<0.001*
	10-12	56	5 (8.9)	51 (91.1)	
	>12	12	7 (58.3)	5 (41.7)	
Vending location	<10	32	24 (75)	8 (25)	0.372
	10-12	56	46 (82.1)	10 (17.9)	
	>12	12	8 (66.7)	4 (33.3)	
Vending cart	<10	32	19 (59.4)	13 (40.6)	0.280
	10-12	56	41 (73.2)	15 (26.8)	
	>12	12	10 (83.3)	2 (16.7)	
Utensils and cutting tools	<10	32	30 (93.8)	2 (6.2)	0.863
	10-12	56	51 (91.1)	5 (8.9)	
	>12	12	12 (100)	0 (0)	
Hygienic practices	<10	32	1 (3.1)	31 (96.9)	<0.001*
	10-12	56	0 (0)	56 (100)	
	>12	12	4 (33.3)	8 (66.7)	
Personal hygiene and habits	<10	32	32 (100)	0 (0)	0.523
	10-12	56	53 (94.6)	3 (5.4)	
	>12	12	12 (100)	0 (0)	
Food preparation, cooking, and handling	<10	32	6 (18.8)	26 (81.3)	0.010*
	10-12	56	17 (30.4)	39 (69.6)	
	>12	12	8 (66.7)	4 (33.3)	
Protection and serving of food	<10	32	11 (34.4)	21 (65.6)	0.009*
	10-12	56	21 (37.5)	35 (62.5)	
	>12	12	10 (83.3)	2 (16.7)	
Handling and disposal of waste	<10	32	0 (0)	32 (100)	-
	10-12	56	0 (0)	56 (100)	
	>12	12	0 (0)	12 (100)	
Pest control	<10	32	0 (0)	32 (100)	-
	10-12	56	0 (0)	56 (100)	
	>12	12	0 (0)	12 (100)	
Training on food safety	<10	32	0 (0)	32 (100)	-
	10-12	56	0 (0)	56 (100)	
	>12	12	0 (0)	12 (100)	

**Table 6:** Relationship between years of experience of the vendors and the various domains of the BIS guidelines BIS: Bureau of Indian Standards Score >50%: satisfactory; score ≤50%: unsatisfactory

Variable	Experience (in years)	Number	Satisfactory N(%)	Unsatisfactory N (%)	p-value (Fisher’s exact test)
Raw material	<4	59	34 (57.6)	25 (42.4)	0.400
	5-9	32	23 (71.9)	9 (28.1)	
	>10	9	6 (66.7)	3 (33.3)	
Transportation, reception, and storage of raw material	<4	59	10 (16.9)	49 (83.1)	0.752
	5-9	32	3 (9.4)	29 (90.6)	
	>10	9	1 (11.1)	8 (88.9)	
Vending location	<4	59	44 (74.6)	15 (25.4)	0.039*
	5-9	32	29 (90.6)	3 (9.4)	
	>10	9	5 (55.6)	4 (44.4)	
Vending cart	<4	59	37 (62.7)	22 (37.3)	0.088
	5-9	32	27 (84.4)	5 (15.6)	
	>10	9	6 (66.7)	3 (33.3)	
Utensils and cutting tools	<4	59	52 (88.1)	7 (11.9)	0.083
	5-9	32	32 (100)	0 (0)	
	>10	9	9 (100)	0 (0)	
Hygienic practices	<4	59	1 (1.7)	58 (98.3)	0.133
	5-9	32	3 (9.4)	29 (90.6)	
	>10	9	1 (11.1)	8 (88.9)	
Personal hygiene and habits	<4	59	59 (100)	0 (0)	0.079
	5-9	32	29 (90.6)	3 (9.4)	
	>10	9	9 (100)	0 (0)	
Food preparation, cooking, and handling	<4	59	13 (22)	46 (78)	0.062
	5-9	32	14 (43.8)	18 (56.2)	
	>10	9	4 (44.4)	5 (55.6)	
Protection and serving of food	<4	59	24 (40.7)	35 (59.3)	0.953
	5-9	32	14 (43.8)	18 (56.3)	
	>10	9	4 (44.4)	5 (55.6)	
Handling and disposal of waste	<4	59	0 (0)	59 (100)	-
	5-9	32	0 (0)	32 (100)	
	>10	9	0 (0)	9 (100)	
Pest control	<4	59	0 (0)	59 (100)	-
	5-9	32	0 (0)	32 (100)	
	>10	9	0 (0)	9 (100)	
Training on food safety	<4	59	0 (0)	59 (100)	-
	5-9	32	0 (0)	32 (100)	
	>10	9	0 (0)	9 (100)	

**Table 7:** Relationship between the place of residence of the vendors and the various domains of the BIS guidelines BIS: Bureau of Indian Standards Score >50%: satisfactory; score ≤50%: unsatisfactory

Variable	Place of residence (local/migrant)	Number	Satisfactory N (%)	Unsatisfactory N (%)	p-value
Raw material	Local	40	26 (65)	14 (35)	0.735 (Chi-square test)
	Migrant	60	37 (61.7)	23 (38.3)	
Transportation, reception, and storage of raw material	Local	40	9 (22.5)	31 (77.5)	0.045 * (Chi-square test)
	Migrant	60	5 (8.3)	55 (91.7)	
Vending location	Local	40	36 (90)	4 (10)	0.025* (Fisher’s exact test)
	Migrant	60	42 (70)	18 (30)	
Vending cart	Local	40	33 (82.5)	7 (17.5)	0.026* (Chi-square test)
	Migrant	60	37 (61.7)	23 (38.3)	
Utensils and cutting tools	Local	40	37 (92.5)	3 (7.5)	1.000 (Fisher’s exact test)
	Migrant	60	56 (93.3)	4 (6.7)	
Hygienic practices	Local	40	3 (7.5)	37 (92.5)	0.386 (Fisher’s exact test)
	Migrant	60	2 (3.3)	58 (96.7)	
Personal hygiene and habits	Local	40	38 (95)	2 (5)	0.562 (Fisher’s exact test)
	Migrant	60	59 (98.3)	1 (1.7)	
Food preparation, cooking, and handling	Local	40	19 (47.5)	21 (52.5)	0.004* (Chi-square test)
	Migrant	60	12 (20)	48 (80)	
Protection and serving of food	Local	40	22 (55)	18 (45)	0.032* (Chi-square test)
	Migrant	60	20 (33.3)	40 (66.7)	
Handling and disposal of waste	Local	40	0 (0)	40 (100)	-
	Migrant	60	0 (0)	60 (100)	
Pest control	Local	40	0 (0)	40 (100)	-
	Migrant	60	0 (0)	60 (100)	
Training on food safety	Local	40	0 (0)	40 (100)	-
	Migrant	60	0 (0)	60 (100)	

**Table 8:** Relationship between the monthly income of the vendors and the various domains of the BIS guidelines BIS: Bureau of Indian Standards Score >50%: satisfactory; score ≤50%: unsatisfactory

Variable	Monthly income (Rupees)	Number	Satisfactory N (%)	Unsatisfactory N (%)	p-value
Raw material	<5000	33	15 (45.5)	18 (54.5)	0.013* (Chi-square test)
	5000-9999	51	39 (76.5)	12 (23.5)	
	≥10000	16	9 (56.3)	7 (43.8)	
Transportation, reception, and storage of raw material	<5000	33	3 (9.1)	30 (90.9)	0.003* (Fisher’s exact test)
	5000-9999	51	4 (7.8)	47 (92.2)	
	≥10000	16	7 (43.8)	9 (56.3)	
Vending location	<5000	33	24 (72.7)	9 (27.3)	0.284 (Chi-square test)
	5000-9999	51	43 (84.3)	8 (15.7)	
	≥10000	16	11 (68.8)	5 (31.3)	
Vending cart	<5000	33	12 (36.4)	21 (63.6)	<0.001* (Fisher’s exact test)
	5000-9999	51	44 (86.3)	7 (13.7)	
	≥ 10000	16	14 (87.5)	2 (12.5)	
Utensils and cutting tools	<5000	33	28 (84.8)	5 (15.2)	0.128 (Fisher’s exact test)
	5000-9999	51	49 (96.1)	2 (3.9)	
	≥ 10000	16	16 (100)	0 (0)	
Hygienic practices	<5000	33	0 (0)	33 (100)	0.141 (Fisher’s exact test)
	5000-9999	51	3 (5.9)	48 (94.1)	
	≥10000	16	2 (12.5)	14 (87.5)	
Personal hygiene and habits	<5000	33	33 (100)	0 (0)	0.407 (Fisher’s exact test)
	5000-9999	51	48 (94.1)	3 (5.9)	
	≥10000	16	16 (100)	0 (0)	
Food preparation, cooking, and handling	<5000	33	2 (6.1)	31 (93.9)	<0.001* (Fisher’s exact test)
	5000-9999	51	19 (37.3)	32 (62.7)	
	≥10000	16	10 (62.5)	6 (37.5)	
Protection and serving of food	<5000	33	8 (24.2)	25 (75.8)	0.004* (Fisher’s exact test)
	5000-9999	51	22 (43.1)	29 (56.9)	
	≥10000	16	12 (75)	4 (25)	
Handling and disposal of waste	<5000	33	0 (0)	33 (100)	-
	5000-9999	51	0 (0)	51 (100)	
	≥10000	16	0 (0)	16 (100)	
Pest control	<5000	33	0 (0)	33 (100)	-
	5000-9999	51	0 (0)	51 (100)	
	≥10000	16	0 (0)	16 (100)	
Training on food safety	<5000	33	0 (0)	33 (100)	-
	5000-9999	51	0 (0)	51 (100)	
	≥10000	16	0 (0)	16 (100)	

The demographic variables were assessed against the overall vendor scores. The compiled data on the demographic variables and overall scores are mentioned in Table 9.

**Table 9:** Relationship between demographic variables and the overall score of the vendors

Variable	Categories	Number	Satisfactory N(%)	Unsatisfactory N(%)	p-value
Age (years)	<25	29	15 (51.7)	14 (48.3)	0.109 (Fisher’s exact test)
	25-34	43	26 (60.5)	17 (39.5)	
	35-44	21	17 (81)	4 (19)	
	≥45	7	6 (85.7)	1 (14.3)	
Gender	Male	95	59 (62.1)	36 (37.9)	0.156 (Fisher’s exact test)
	Female	5	5 (100)	0 (0)	
Level of education	<10	32	21 (65.6)	11 (34.4)	0.693 (Fisher’s exact test)
	10-12	56	34 (60.7)	22 (39.3)	
	>12	12	9 (75)	3 (25)	
Experience	<4 yrs	59	35 (59.3)	24 (40.7)	0.538 (Fisher’s exact test)
	5-9 yrs	32	23 (71.9)	9 (28.1)	
	≥10 yrs	9	6 (66.7)	3 (33.3)	
Residence	Local	40	33 (82.5)	7 (17.5)	0.002* (Chi-square test)
	Migrant	60	31 (51.7)	29 (48.3)	
Income (Rs/month)	<5000	33	15 (45.5)	18 (54.5)	0.023* (Chi-square test)
	5000-9999	51	38 (74.5)	13 (25.5)	
	≥10000	16	11 (68.7)	5 (31.3)	

## Discussion

During the study, it was observed that the mean age of the vendors was 30.47 ± 8.06 years, with an average experience in food vending of 4.87 ± 2.93 years. These findings contrast with a study conducted in Kolkata by Mukherjee et al., which reported an average age of 37 ± 10.7 years and a mean vending experience of 13.37 ± 8.06 years [14]. However, the results were comparable to the study conducted on street food vendors in Lahore, Pakistan, by Ahmed et al., where 79.2% of vendors were between the ages of 19 and 35 years, and 59.4% of vendors had experienced between one and five years [15]. In our study, it was found that 92% of the vendors had an experience of fewer than 10 years, which was comparable to other studies conducted in Hyderabad by Reddi et al. and in Guwahati by Choudhury et al., where the majority of the vendors had the experience of food vending of fewer than 10 years [16,17]. The results were consistent with a study in Southern Ethiopia by Negassa et al., where the average experience of street food vendors was three years [18].

In a study conducted in Hyderabad, India, by Reddi et al., all the study participants were males. In our study, 95% of the respondents were males, while only 5% were females, which was comparable [16]. Similar findings were also noted in a study conducted in Chandigarh by Singh et al., where 93% of the vendors were males and 7% were females [19]. However, the findings were in contrast to the study conducted by Negassa et al. in Southern Ethiopia, in which 65.9% of vendors were females [18].

This study found that 40% of the vendors were local residents, while 60% were migrants. This finding is similar to that of a study conducted in Noida, Uttar Pradesh, by Singh et al., which reported that 45% of the vendors were migrants [20]. However, this differed from the study conducted in Assam by Choudhury et al., where 93% of the vendors were locals of Guwahati City [17].

In our study, we found that 32% of the vendors had education up to the secondary level, equivalent to the 10th standard, and 68% had education beyond the secondary level. These findings align with a study conducted by Reddi et al. in Hyderabad, India, where 30% of the vendors had completed their secondary education [16]. In our study, we found a statistically significant relationship between the level of education and the hygiene practices of street food vendors (a domain in the questionnaire), where more educated vendors had better practices. Similar results were observed in a study conducted in Agartala by Reang et al., which also reported a significant relationship between the level of education and the hygiene practices of vendors [21]. However, in the same study, it was found that vendors, regardless of their level of education, were generally unaware of the importance of washing their hands before serving food.

In the present study, the monthly income of street food vendors varied from Rs. 1200 to Rs. 15000, with a mean income of Rs. 6004 ± 3179. This is comparable to a study conducted in Guwahati, Assam, by Choudhury et al., where vendors earned between Rs. 200 and Rs. 600 per day, resulting in a monthly income ranging from Rs. 6000 to Rs. 18000 [17]. The study found that 64% of the vendors demonstrated satisfactory practices. This result aligns with a meta-analysis conducted by Desye et al., which revealed that 51% of the vendors exhibited good vending practices [22].

In our study, we did not find a statistically significant relationship between the level of education and the overall score of street food vendors. This result is similar to that of a study conducted by Okojie et al. in Benin City, Nigeria [23].

A study conducted in Lahore, Pakistan, found that demographic variables such as the age of vendors, education level, and experience were significantly related to their knowledge, attitudes, and practices (KAP) regarding food safety. The only demographic variable that did not show a significant relationship with KAP of food hygiene in this study was gender [15]. In our study, we found that age, gender, level of education, and experience did not have a significant association with the food safety and hygiene practices of street food vendors. However, we discovered that both place of residence and income were significantly related to these practices. A meta-analysis conducted by Desye et al. found that street vendors with higher incomes were more likely to practice better hygiene [22]. Our study observed similar findings, revealing a significant relationship between vendors' income and their hygiene practices.

Our study found that the lack of waste disposal facilities contributed to the unhygienic practices of street food vendors. This finding was also supported by a newspaper article that reported that food stalls on the roadside were leading to an increase in litter and filth in those areas [24]. In conditions like this, the food sold by vendors is highly likely to cause foodborne illnesses, especially diarrheal diseases.

In our study, it was observed that there are two main heads under which the factors for nonconformance of food safety and hygiene practices can be divided: (1) the responsibility of vendors and (2) the responsibility of stakeholders. Factors such as maintaining basic hygiene like handwashing, covering hair during food preparation, proper cleaning of utensils, etc., come under the responsibility of the vendors. Factors such as providing earmarked places, adequate lighting, waste disposal from the site of vending food, medical examination of vendors, provision of water, etc., are the prerogative of the stakeholders like a municipal corporation, water and electricity department, health department, etc. There are some grey areas also where the vendors and stakeholders have to come in tandem to solve issues like washing hands and utensils; this can only be done if vendors are sensitive about the issue and have water facilities at the location. These factors were also brought out in a study conducted in Southern Ethiopia, in which the lack of clean, appropriate water and sanitation was identified as a factor in improving the quality of street-vended food. This study also emphasized the general improvement of hygiene and sanitation of the area [18].

During the study, it was observed, as well as told by the vendors, that waste disposal facilities are not available at the site of the vending location. This resulted in unsanitary waste disposal, creating favorable conditions for the breeding of rodents and vermin. These findings were also corroborated in a study conducted in Alexandria by Koraish et al., where the lack of waste disposal facilities was an important factor in the unhygienic conditions of street food vending [25]. Improper waste disposal, inadequate water supply, and unhygienic surroundings like sewage also provide breeding sites for flies and mosquitoes [24].

The strengths of this study include its novelty, where the BIS food safety requirements were converted into the questionnaire. These standards have not previously been used to assess the food safety and hygiene practices of street food vendors. The study has a limitation in that it was carried out in an urban slum of Pune, Maharashtra, so the results cannot be generalized to the other parts of the country. Therefore, similar studies in other regions of the country are necessary to evaluate the compliance of street food vendors with the BIS. 

## Conclusions

A total of 64% of the street vendors in the area achieved satisfactory scores with respect to the questionnaire developed from the BIS guidelines on street food vendors' food safety requirements. Nevertheless, there remains significant room for improvement, particularly in the areas of reception, transportation, and storage of raw materials, waste disposal, pest control, and vendor training. Enhancing these parameters requires the involvement of various stakeholders. Local government authorities can play a crucial role by designating specific areas for street food vending, providing proper waste disposal facilities, offering pest control support, and conducting training programs for vendors. The health department can contribute by educating vendors on hygiene and sanitation practices and carrying out regular medical examinations of the vendors. The law enforcement department can elevate standards by enforcing registration and rigorously implementing guidelines. Finally, the water and electricity departments can assist by supplying electricity and safe, potable water to vendors at designated vending sites. An effective method for enhancing the hygiene and sanitation standards of street food vendors involves providing them with smart skill and registration cards following appropriate training. A comparable project was initiated in Bangkok in 1994, resulting in enhanced standards for street foods.

Established in 1998, the National Association of Street Vendors of India (NASVI) was created to address the challenges faced by street vendors. The Street Vendors (Protection of Livelihood and Regulation of Street Vending) Act, enacted in 2014, seeks to unify various stakeholders to effectively manage and overcome the issues related to street vending. The Government of India, through the Ministry of Skill Development and Entrepreneurship, initiated a program in 2021 aimed at training street food vendors. The NASVI, in partnership with national and local food authorities such as the Food Safety and Drug Administration, Uttar Pradesh, and the Food Safety and Standards Authority of India (FSSAI), has launched Project "Serve Safe Food," which aims to train street food vendors. Such projects are bringing together various stakeholders, which can improve the food safety and hygiene practices of street food vendors.

Studies should also be conducted in other regions of the country to assess the status of food safety and hygiene practices of street food vendors and the implementation of corrective measures by the stakeholders, if needed.

## Appendices

                                                                Queastionnaire

**Table 10:** Questionnaire BIS: Bureau of Indian Standards

	As per BIS						
Domain							
Raw material	Unsatisfactory (0)			Fair (1)		Satisfactory (2)	
Fresh							
Dry							
Transportation, reception, and storage of raw materials							
	No cold chain (0)			Cold chain maintained partially (1)		Cold chain completely (2)	
Temperature for items requiring refrigeration							
	Percentage of containers						
	Less than 25% (0)		25%-50% (1)		50%-75% (2)		More than 75% (3)
Condition of containers which were hygienic							
Labelling of containers							
	Unsatisfactory (0)				Satisfactory (1)		
Storage of non-food items							
Disposal of waste material							
	No (0)				Yes (1)		
Separate storage of raw and cooked food							
Separate storage of fuel							
Vending location							
	Less than 15 mts (0)				More than 15 mts (1)		
Away from rubbish							
Away from toilet							
Away from open drains							
Away from wastewater							
	Present (0)				Absent (1)		
Interference with vehicular traffic							
Obstruction to pedestrians							
	No (0)				Yes (1)		
Sale point surrounding clean and litter free							
Sale point surrounding free of animals and pets							
Adequate natural/artificial lighting							
Wastewater disposal facilities provided							
Rubbish disposal facilities provided							
Container for waste material specifically identifiable							
Rubbish bin covered							
Rubbish bin made of impermeable material							
Rubbish bin easy to clean							
Rubbish bin provided with a plastic bag inside							
Vending cart							
Hygienic							
Impermeable							
Easy to clean working surface( like stainless steel							
At least 60 to 70 cms above ground							
Sale point							
Awnings							
Glass boxes							
Vending cart built of solid materials							
Vending cart built rust/corrosion resistant materials							
Vending cart kept in good condition							
		Absent (0)			Present (1)		
Transported drinking water in protected containers of at least 20 ltrs							
Vending cart protected from sun							
Vending cart protected from dust							
Vending cart protected from wind							
Food vending cart kept in clean place when not in use.							
Sale points/vans/carts free of any personal clothing							
Utensils and Cutting tools							
Cooking utensils easy to clean							
Cooking utensils corrosion resistant							
Cooking utensils and crockery clean							
Cooking utensils and crockery not broken/chipped							
Utensils not wiped with unclean cloth							
Cooking not done in utensils of copper, cadmium, lead, non-food grade plastic and other toxic material							
storage not done in utensils of copper, cadmium, lead, non-food grade plastic and other toxic material							
serving not done in utensils of copper, cadmium, lead, non-food grade plastic and other toxic material							
Utensils cleaned of debris after every operation							
Utensils scrubbed with detergent after every operation							
Utensils washed under running water after every operation							
Cleaned utensils air dried							
Utensils stored in a protected place							
Hygienic Practices							
		No (0)			Yes (1)		
Food handlers wash hands with soap and water before handling food							
Utensils used to serve food washed before putting back into pot							
Fingers kept away from rims of cups, glasses, plates and dishes.							
Ready to eat food or ice handled with utensils like scoops, spoons, spatulas, tongs, ladles, paper napkins and disposable hand gloves							
Handles of scoops, spoons, spatulas, tongs, ladles etc kept out of food/ice to be handled							
Food handlers/ consumers hold cutlery by handles only							
Hand gloves, if used, are disposable							
Gloves discarded during interruptions like visiting toilets, resting							
Bare hand handling ready to eat food not used							
Hands washed after handling money before handling food again							
Hands washed before putting on gloves							
Food handlers wear head cover							
Food handlers wear aprons							
Food handlers wear beard cover							
Separate container with tap available for hand wash							
Personal Hygiene and habits							
		Absent (0)			Present (1)		
Food handlers free from infectious diseases							
Food handlers do not sneeze/cough directly over food							
Food handlers refrain from smoking during preparation and serving of food							
Food handlers observe elementary personal hygiene habits like clean, short finger nails							
clean hands							
covering hair during food handling							
Food handlers do not wear rings during food handling							
Food handlers do not wear bracelets, wrist watch during food handling							
Food handlers do not handle food with skin problems							
Food handlers do not handle food with GI problems							
Food preparation, Cooking and handling							
		Absent (0)			Present (1)		
Cooked food and potentially hazardous food kept in cool well ventilated place or at temperatures <5 degree centigrade							
Cooked food reheated once only in the portion to be served							
Use of repeatedly heated vegetable oil avoided							
Food cooked/ kept outdoors protected against dust							
Food cooked/ kept outdoors protected against sun							
Greens and other vegetables washed with potable water							
Food like rice, pulses or mat washed before preparation with running drinking water							
Protection and Serving of food							
Food prepared for the day used on the same day and not served the next day							
Use of serving utensils like tongs, spoons etc for serving food							
Take away food wrapped in fresh food grade paper/plastic/aluminium foil							
Left over portions of the food by the customers not served again except for unopened packaged food							
Separate utensils used for each type of food							
Food stored at appropriate temperature in fridge/freezer							
Person serving food wore disposable food grade gloves							
Disposables used only once							
Reusable plates kept clean and in good condition							
Container lids kept clean and in good condition							
Glasses kept clean and in good condition							
Disposable plates are used							
Container lids are used							
Disposable Glasses are used							
Handling and Disposal of Waste							
Rubbish bins kept covered away from the place where food is handled							
Rubbish bins with foot operated lids							
		Absent (0)			Present (1)		
Solid and liquid waste kept separately							
Liquid waste disposed in the nearest drain							
Pest Control							
Food vending area kept clean and tidy							
Food vending area fumigated periodically with approved chemicals							
Training							
Vendor or food handler underwent basic training in food hygiene before starting street food vending							
